# Merkel Cell Carcinoma of Unknown Primary: Immunohistochemical and Molecular Analyses Reveal Distinct UV-Signature/MCPyV-Negative and High Immunogenicity/MCPyV-Positive Profiles

**DOI:** 10.3390/cancers13071621

**Published:** 2021-03-31

**Authors:** Piotr Donizy, Joanna P. Wróblewska, Dora Dias-Santagata, Katarzyna Woznica, Przemyslaw Biecek, Mark C. Mochel, Cheng-Lin Wu, Janusz Kopczynski, Malgorzata Pieniazek, Janusz Ryś, Andrzej Marszalek, Mai P. Hoang

**Affiliations:** 1 Department of Pathomorphology and Oncological Cytology, Wroclaw Medical University, 50-556 Wroclaw, Poland; piotrdonizy@wp.pl; 2 Department of Pathology, Poznan University Medical Sciences and Greater Poland Cancer Center, 61-866 Poznan, Poland; Joanna.wroblewska@wco.pl (J.P.W.); amars@ump.edu.pl (A.M.); 3 Department of Pathology, Massachusetts General Hospital and Harvard Medical School, Boston, MA 02114, USA; ddiassantagata@mgh.harvard.edu; 4 Department of Mathematics and Information Science, Warsaw University of Technology, 00-6628 Warsaw, Poland; Woznicakatarzyna22@gmail.com (K.W.); przemyslaw.biecek@gmail.com (P.B.); 5 Department of Pathology, Virginia Commonwealth University, Richmond, VA 23298, USA; Mark.Mochel@vcuhealth.org; 6 Department of Pathology, National Cheng Kung University Hospital, College of Medicine, National Cheng Kung University, Tainan 70403, Taiwan; wujl.towalkwithwings@gmail.com; 7 Department of Surgical Pathology, Holy Cross Cancer Centre, 25-734 Kielce, Poland; janusz.kopczynski@onkol.kielce.pl; 8 Department of Oncology and Division of Surgical Oncology, Wroclaw Medical University, 530-413 Wroclaw, Poland; malgorzatapieniazek@interia.pl; 9 Department of Pathology, Maria Sklodowska-Curie National Research Institute of Oncology, 31-115 Cracow Branch, Poland; z5rys@cyf-kr.edu.pl

**Keywords:** Merkel cell carcinoma, unknown primary, Merkel cell polyomavirus, *TP53*, *PIK3CA*, UV signature, TdT, Pax5, p53, Rb

## Abstract

**Simple Summary:**

Merkel cell carcinomas (MCCs) of unknown primary are defined as deep-seated tumors without an associated cutaneous tumor. Although the distinction has important clinical implications, it remains unclear whether these tumors represent primary tumors of lymph nodes or metastatic cutaneous primaries. We compared the immunohistochemical profiles of four groups of Merkel cell carcinomas (virus-positive and virus-negative unknown primary tumors and virus-positive and virus-negative cutaneous tumors) and performed molecular studies on the unknown primary tumors. Virus-positive and virus-negative Merkel cell carcinomas of unknown primary (MCC-UPs) exhibited an immunoprofile similar to virus-positive and virus-negative primary cutaneous MCCs, respectively. Similar to primary cutaneous Merkel cell carcinomas, virus-negative unknown primary tumors exhibited UV signatures and frequent high tumor mutational burdens, whereas few molecular alterations were noted in virus-positive tumors. Although additional studies are warranted for the virus-positive cases, our findings are supportive of a cutaneous metastatic origin for virus-negative Merkel cell carcinomas of unknown primary.

**Abstract:**

Background: Merkel cell carcinomas of unknown primary (MCC-UPs) are defined as deep-seated tumors without an associated cutaneous tumor. Although the distinction has important clinical implications, it remains unclear whether these tumors represent primary tumors of lymph nodes or metastatic cutaneous primaries. Methods: We compared the immunohistochemical profiles of four groups of MCCs (Merkel cell polyomavirus (MCPyV)-positive UP, MCPyV-negative UP, MCPyV-positive known primary (KP), and MCPyV-negative KP) using B-cell and pre-B-cell markers, cell cycle regulating proteins, follicular stem cell markers, and immune markers, and performed next generation and Sanger sequencing. Results: Virus-positive and virus-negative MCC-UPs exhibited an immunoprofile similar to virus-positive and virus-negative primary cutaneous MCCs, respectively. MCC-UP tumors (both virus-positive and -negative) were immunogenic with similar or even higher tumoral PD-L1 expression and intratumoral CD8 and FoxP3 infiltrates in comparison to MCPyV-positive cutaneous tumors. In addition, similar to primary cutaneous MCCs, MCPyV-negative MCC-UPs exhibited UV signatures and frequent high tumor mutational burdens, whereas few molecular alterations were noted in MCPyV-positive MCC-UPs. Conclusions: Our results showed distinct UV-signatures in MCPyV-negative tumors and high immunogenicity in MCPyV-positive tumors. Although additional studies are warranted for the MCPyV-positive cases, our findings are supportive of a cutaneous metastatic origin for MCPyV-negative MCC-UP tumors.

## 1. Introduction

Merkel cell carcinoma (MCC) is a rare, aggressive neuroendocrine tumor with high mortality (33–46%) [1,2,3,4,5]. MCC is more common in men and in Caucasians [4,6,7]. Risk factors for developing MCC include exposure to ultraviolet (UV) radiation, older age, and immunosuppressive conditions such as human immunodeficiency virus (HIV) infection, organ transplantation, and chronic lymphocytic leukemia [8]. Currently there are two hypothesized pathways in MCC pathogenesis: (1) clonal integration of the Merkel cell polyomavirus (MCPyV) into the dermal-located precursor cells, and (2) ultraviolet irradiation of the intraepidermal stem cells or Merkel cells, which is characterized by a UV-mutational signature: frequent C > T/A > G and CC > TT/AA > GG transitions and recurrent tumor protein 53 (*TP53*) and RB transcriptional corepressor 1 (*RB1*) mutations [9,10]. 

Approximately 4% of all MCCs present as deep-seated tumors in the parotid, axillary, or inguinal region with no overlying skin involvement or a history of primary tumor [11]. MCC of unknown primary (MCC-UP) affects predominantly white elderly men with recurrences noted in a third of the patients [12]. Interestingly, MCC-UPs have been shown to have improved survival compared to those with similar stage and an identifiable concurrent primary tumor [13,14]. Due to the expression of critical early B-cell markers, including TdT, Pax5, and CD117, in a subset of MCC [9,10], MCC-UP has been proposed to be a primary lymph node tumor at these regions deriving from pre- or pro-B cells. Recent studies reported abundant UV-signature mutations, suggesting MCC-UP to be a nodal metastasis from regressed MCCs (via enhanced immune function) from sun-exposed skin rather than a metastasis from extracutaneous sites or nodal primaries [15,16].

Despite a clear molecular delineation between the MCPyV-positive and MCPyV-negative cutaneous MCCs, little is known about the biology of MCC-UP. The distinction between whether these tumors represent primary tumors or metastases is important for the development of relevant preclinical models and would affect treatment strategy. In order to investigate the potential cell-of-origin and possible molecular therapeutic targets, we compared the immunohistochemical profiles of the four groups of MCCs (MCPyV-positive UP, MCPyV-negative UP, MCPyV-positive known primary (KP), and MCPyV-negative KP) using B-cell and pre-B-cell markers (Pax5, TdT), cell cycle regulating proteins (p53, Rb), follicular stem cell markers (CK15, CK19), and immune markers (PD-L1, IDO1, CD8, FoxP3). We also analyzed the molecular profiles of MCPyV-positive UPs versus MCPyV-negative UPs by next generation sequencing (NGS) and targeted Sanger sequencing.

## 2. Results

### 2.1. MCC-UPs Exhibited Better Overall Survival in Comparison to the Virus-Negative KP Group. MCPyV-Negative UPs with High Intratumoral CD8+ and Foxp3+ Infiltrate Exhibited Better Overall Survival

The ages of the 27 patients (22 males, 5 females) with MCC-UPs ranged from 49 to 95 years (median, 70 years). Although a significant history of immunosuppression was not noted in these patients, comorbidities of prostatic carcinoma and melanoma were documented in three and four patients, respectively. The tumor was within lymph nodes of the parotid gland (15 cases), the pelvic/groin region (7 cases), the submandibular gland (4 cases), and the axilla (1 case). Their sizes ranged from 1.0 to 9 cm (median, 2.5 cm). Overall, 6 patients received surgery, 6 received surgery and radiation, and 15 received surgery, radiation, and chemotherapy (12 with carboplatin/etoposide and 3 with pembrolizumab). Follow-ups ranged from 0 to 201 months (median, 27 months). Death was documented in 13/27 (48%) of patients. The immunohistochemical results of MCC-UP cases versus MCPyV status are summarized in Supplemental Table S1. MCPyV-negative UP cases exhibited significantly higher p53 (*p* = 0.037) and CK15 (*p* = 0.014) expression than MCPyV-positive UP cases.

The clinicopathologic variables of the primary cutaneous MCCs are summarized in Supplemental Table S2. Sixty-three percent (85/134) of tumors were positive for CM2B4. The age of the 134 patients (75 males, 59 females) ranged from 52 to 94 years (median, 77 years). Immunosuppression was noted in 13 patients (10%). At diagnosis, 68 patients were classified as stage I, 53 as stage II, 12 as stage III, and none as stage IV. The range of follow-ups for all patients was 0 to 255 months (median, 22 months). Local recurrence and/or metastasis (progression) developed in 55/134 (41%) of patients (recurrence in 7, metastasis in 35, both recurrence and metastasis in 13 patients). Death was documented in 74/134 (55%) of patients.

In total, 64 tumors (48%) were from the head and neck region and 70 (52%) were from other sites. The median tumor size and tumor thickness were 20 mm (range: 2 to 125 mm) and 10 mm (range: 1 to 55 mm), respectively. Mitoses per squared millimeter ranged from 1 to over 100 (median, 40). Ulceration, necrosis, perineural invasion, and lymphovascular invasion were present in 45 (34%), 44 (33%), 13 (10%), and 64 (48%) cases, respectively. The presence of epidermotropism (*p* = 0.0002) and associated keratinocytic neoplasms (*p* = 0.0001) was significantly correlated with MCPyV-negative status.

**Figure 1:**
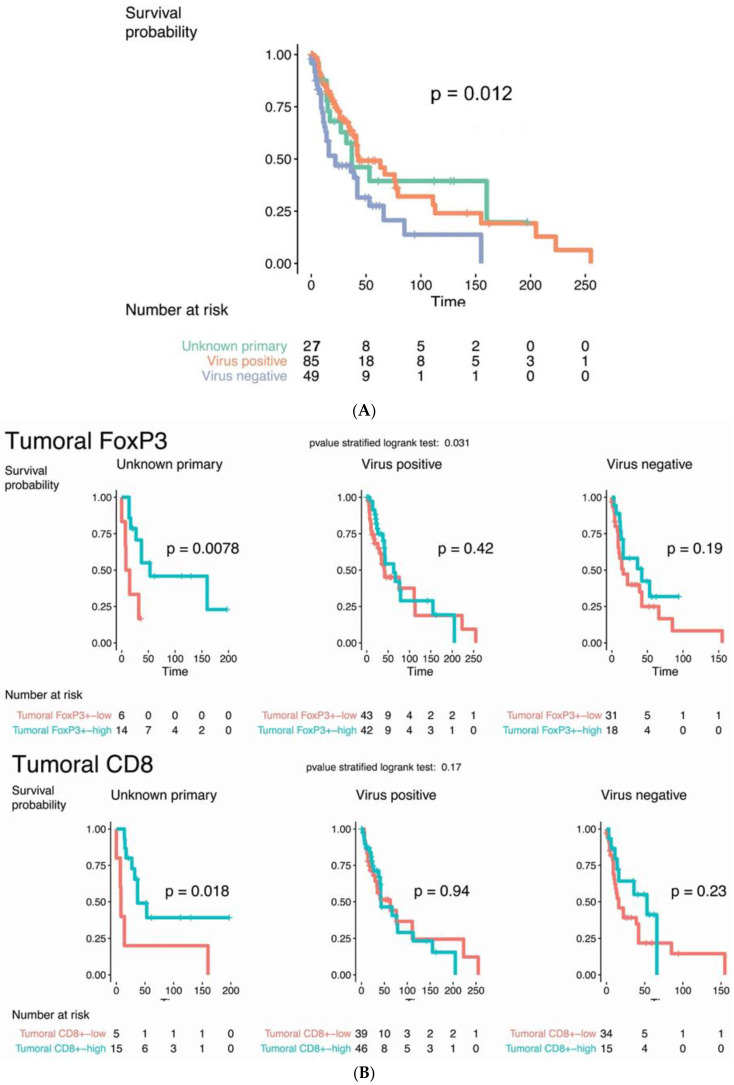
(**A**) Kaplan–Meier curves of overall survival in the three groups (*p* = 0.012). (**B**) Kaplan–Meier curves demonstrate better overall survival in Merkel cell carcinoma of unknown primary with high intratumoral FoxP3+ (*p* = 0.0078) and high intratumoral CD8+ (*p* = 0.018) infiltrates. Significant correlations were not seen in the virus-positive and virus-negative groups of known primary.

**Figure 2:**
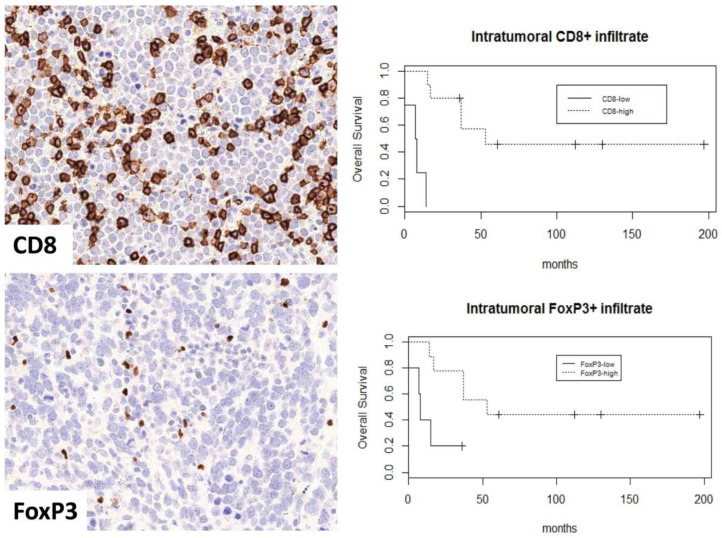
Kaplan–Meier curves demonstrate better overall survival in MCPyV-negative Merkel cell carcinoma of unknown primary with high intratumoral CD8+ (*p* < 0.0001) infiltrate and high intratumoral FoxP3+ (*p* = 0.026) infiltrate.

Kaplan–Meier curves demonstrated significant differences in the overall survival (OS) among the three groups (UP, virus-positive KP, and virus-negative KP) (*p* = 0.012) with the worst survival noted in the virus-negative KP group (Figure 1A). Kaplan–Meier curves of OS in MCC-UPs versus stage III primary cutaneous MCCs demonstrated no significant survival difference (*p* = 0.44). MCC-UP tumors with high intratumoral FoxP3+ and CD8+ infiltrates exhibited better OS (*p* = 0.0078 and 0.018, respectively) (Figure 1B). When only virus-negative UP cases were analyzed, high intratumoral CD8+ and FoxP3+ infiltrates remained predictors of improved OS (log-rank *p*-values < 0.0001 and 0.026, respectively) (Figure 2), whereas CD8+ and FoxP3+ infiltrates had no prognostic significance in virus-positive MCC-UPs (log-rank *p*-values = 0.31 and 0.74, respectively). There was no survival difference observed in the MCC-UP group with respect to MCPyV status, PD-L1, IDO1, and TdT (*p* = 0.93, 1, 0.36, 0.2, respectively). No significant associations between intratumoral FoxP3+ infiltrate (*p* = 0.42 and 0.19) and CD8+ infiltrate (*p* = 0.94 and 0.23) versus OS were observed in virus-positive and virus-negative KP groups, respectively.

### 2.2. Virus-Positive and Virus-Negative MCC-UPs Exhibited an Immunoprofile Similar to Virus-Positive and Virus-Negative Cutaneous MCCs, Respectively

**Figure 3:**
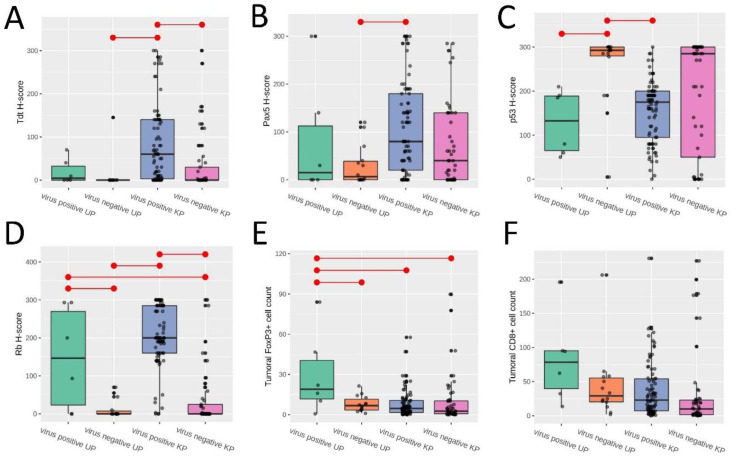
A comparison of the four groups: Merkel cell polyomavirus (MCPyV)-positive unknown primary (UP); MCPyV-negative UP; MCPyV-positive known primary (KP); and MCPyV-negative KP. (**A**) A box plot of TdT nuclear *H*-score. The box is limited by the 25th and 75th percentiles, and the black bar represents the median line. Connecting bars indicate statistical significance between groups. Boxplots of the (**B**) Pax5 nuclear *H*-score, (**C**) p53 nuclear *H*-score, (**D**) Rb nuclear *H*-score, (**E**) intratumoral FoxP3+ cell count, and (**F**) intratumoral CD8+ cell count.

The immunohistochemical expression was compared among the four groups by box-plot analyses and the *p*-values with false discovery rate corrections are summarized in Supplemental Table S3. TdT expression was significantly lower and Pax5 expression was approaching significantly lower in the virus-negative UP group versus the virus-positive KP group (*p* = 0.026 and 0.088, respectively) (Figure 3A,B). Nuclear p53 expression was higher in virus-negative UPs versus virus-positive KPs (*p* = 0.0096) (Figure 3C). Rb expression was higher in virus-positive UPs versus virus-negative UPs and virus-negative KPs (*p* = 0.0088 and 0.016, respectively), and in virus-positive KPs versus virus-negative UPs and virus-negative KPs (*p* ≤ 0.0001 and <0.0001, respectively) (Figure 3D). There was no significant difference in CK15 and CK19 expression among the four groups. 

### 2.3. Virus-Positive and Virus-Negative MCC-UPs Exhibited Tumoral Immune Expression Similar to or Higher Than That of Virus-Positive MCC-KPs

**Figure 4:**
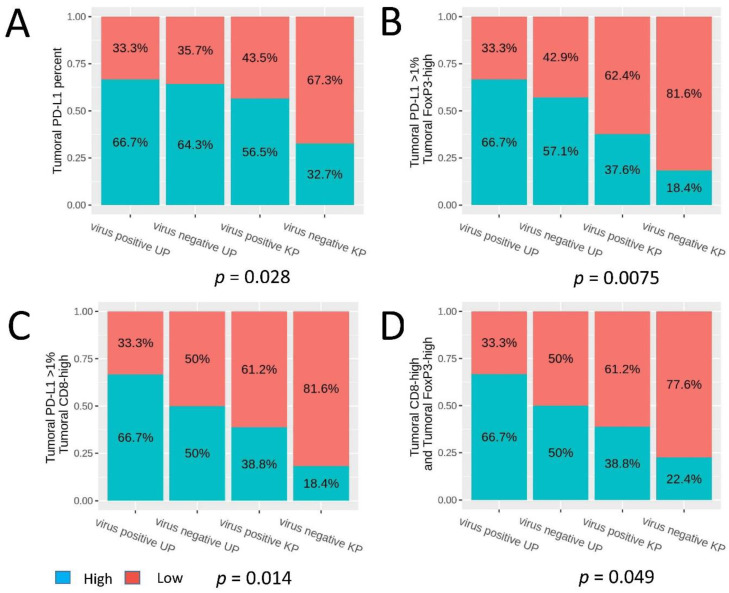
Comparisons of (**A**) tumoral PD-L1 expression and high coexpression of (**B**) PD-L1 and CD8, (**C**) PD-L1 and FoxP3, and (**D**) CD8 and FoxP3 in the four groups: MCPyV-positive unknown primary (UP); MCPyV-negative UP; MCPyV-positive known primary (KP); and MCPyV-negative KP.

The FoxP3+ cell count was significantly higher in the virus-positive UP group versus the virus-negative UP, virus-positive KP, and virus-negative KP groups (*p* = 0.017, 0.009, and 0.017, respectively) (Figure 3E). Although there was no significant difference in the intratumoral CD8+ cell count between the four groups, similar median counts were observed between the virus-negative UP and virus-positive KP groups (Figure 3F). We observed a trend of a higher combined tumoral PD-L1 expression with high intratumoral FoxP3+ infiltrate and tumoral PD-L1 expression with high intratumoral CD8+ infiltrate in the virus-negative UP versus the virus-negative KP groups (*p* = 0.066 and 0.081, respectively). A sequential decrease in tumoral PD-L1 expression, combined tumoral PD-L1 and high intratumoral FoxP3+ cell count, combined tumoral PD-L1 and high intratumoral CD8+ cell count, and combined high intratumoral CD8+ and FoxP3+ cell counts was observed in the virus-positive UP, virus-negative UP, virus-positive KP, and virus-negative KP groups (chi-square *p*-values = 0.028, 0.00075, 0.014, and 0.049, respectively) (Figure 4).

### 2.4. Virus-Positive and Virus-Negative MCC-UPs Exhibited Molecular Alterations Similar to Virus-Positive and Virus-Negative Primary Cutaneous MCCs, Respectively

**Figure 5:**
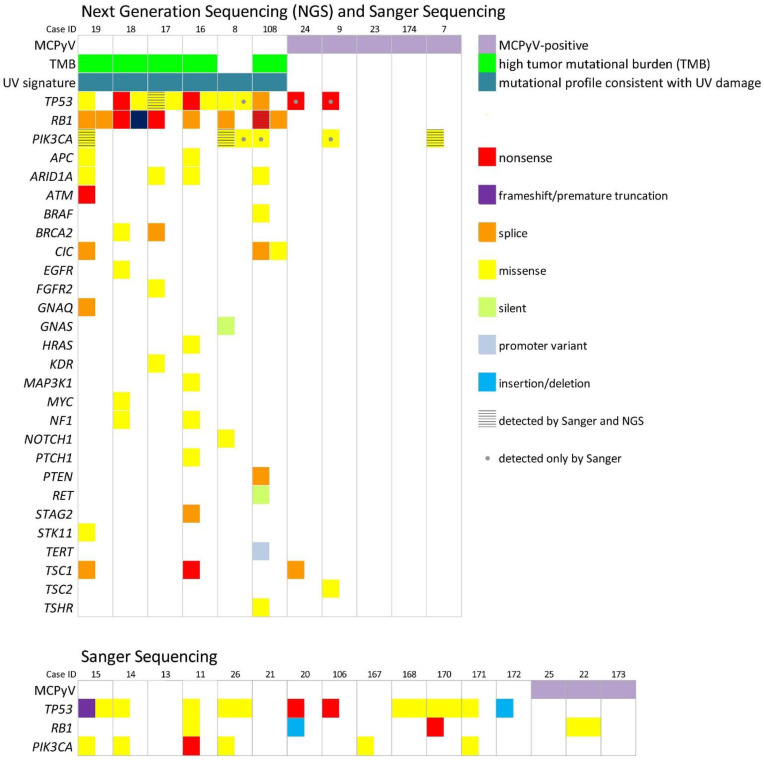
A summary of the next generation sequencing and Sanger sequencing results. Mutations including *TP53*, *RB1*, and *PIK3CA* are more frequently noted in MCPyV-negative versus MCPyV-positive unknown primaries. By next generation sequencing analyses, *TP53* and *RB1* mutations and/or insertions/deletions with C > T/G > A and CC > TT/GG > AA transitions and high tumor mutational burdens were detected in all six and five virus-negative MCC-UPs, respectively. These findings are supportive of a UV-induced etiology.

The results of the molecular analyses are summarized in Supplemental Table S4. By NGS analyses, *TP53* and *RB1* mutations and/or insertions/deletions with C > T/G > A and CC > TT/GG > AA transitions (CC > TT highly suggestive of UV-induced mutation) were detected in all six virus-negative MCC-UPs (Figure 5). C > T/ G > A transitions were noted in other genes including *PIK3CA*, *ARID1A*, *APC*, *ATM*, *BRAF*, *BRCA2*, *CIC*, *EGFR*, *GNAQ*, *GNAS*, *MAP3K1*, *NOTCH1*, *PTEN*, *RET*, *STAG2*, and the *TERT* promoter region (Supplemental Table S4). In addition, five of the six virus-negative MCC-UPs exhibited high tumor mutational burden (TMB). These findings are supportive of a UV-induced etiology. On the contrary, only rare mutations involving *TSC1* and *TSC2* were detected in the five virus-positive MCC-UPs. By Sanger sequencing, *TP53*, *RB1*, and *PIK3CA* mutations were more frequently detected in virus-negative MCC-UPs than virus-positive MCC-UPs (Figure 5). These findings mirror those previously reported in primary cutaneous MCCs: mutations of the *TP53*, *RB1*, and *PIK3CA* genes in 81%, 69%, and 29% of MCPyV-negative tumors, respectively, and in 3.7%, 2.4%, and 1.7% of MCPyV-positive ones, respectively [17,18,19,20,21].

## 3. Discussion

Although MCC-UP has been considered by some to be a primary lymph node tumor, in our study, virus-negative and virus-positive MCC-UPs demonstrated immunoprofiles and molecular alterations that mirror those of virus-negative and virus-positive primary cutaneous MCCs, which is highly suggestive of a cutaneous metastatic origin. MCPyV-positive and -negative cutaneous MCCs have been shown to have different clinical behaviors, histologic features, and immunoprofiles. In line with our results, several previous studies have demonstrated that MCPyV-positive MCCs have a more favorable prognosis than MCPyV-negative MCCs [22,23,24,25]. 

The cellular origin of MCC remains unclear. The varying histologic features observed between MCPyV-positive and -negative MCCs support a different histogenesis. In our study, the presence of epidermotropism and associated keratinocytic neoplasms, and the high expression of CK15 (follicular stem cell marker) in cutaneous MCC, were significantly correlated with an MCPyV-negative status [26,27]. These results support the hypothesis that MCPyV-negative MCCs are derived from intraepithelial, possibly follicular stem cells affected by UV-induced oncogenesis. The histogenesis of MCPyV-positive MCCs differs, with dermal and subcutaneous tumors usually sparing the epidermal and follicular epithelium [26]. In more than 80% of cases, clonal integration of MCPyV into the host genome has been observed [28]. Pre-/pro-B cells have been proposed to be the precursors of these MCPyV-positive tumors due to the expression of TdT, Pax5, and various immunoglobulins [10]. However, dermal fibroblasts and not pre-/pro-B cells have been shown to support MCPyV infection in vitro [29]. Therefore, the precursor cells of MCPyV-positive MCC-UPs remain uncertain. They might be dermal stem cells in the setting of cutaneous metastases or nodal B-precursor cells if MCC-UP represents a lymph node primary [10].

In our series, virus-positive and virus-negative MCC-UPs exhibited an immunoprofile similar to virus-positive and virus-negative cutaneous MCCs, respectively, which is highly suggestive that UP tumors represent metastases of cutaneous primary. The overexpression of TdT and Pax5 as well as the up-regulation of Rb protein has been shown to significantly correlate with MCPyV positivity in cutaneous MCCs [27]. On the other hand, p53 expression is associated with MCPyV-negative MCCs [22,30]. Similar to virus-negative MCC-KP, virus-negative MCC-UP tumors in our study exhibited an immunoprofile characterized by low TdT, low Pax5, high p53, and absent/low Rb expression. Our study showed significant differences in the levels of TdT and Pax5 expression between virus-negative MCC-UPs and virus-positive MCC-KPs. Similarly, high p53 and high RB expression were noted in the virus-negative and virus-positive groups (UP and KP), respectively.

A strong immune response has been shown to correlate with a better outcome for MCC patients [23]. Several previous studies have reported that intratumoral CD8+ lymphocytes are strongly correlated with better survival [23,31,32,33,34]. Similarly, MCC-UP tumors with high intratumoral CD8+ and FoxP3+ infiltrates exhibited improved OS in our study. Although a prognostic association was not noted for CD8 when subgroups (virus-positive KP and virus-negative KP) were analyzed in the current study, a CD8+ infiltrate correlated with improved MCC-specific survival (*p* = 0.036) when the entire primary cutaneous MCC group (virus-positive and virus-negative KP) was analyzed (unpublished data). While the CD8+ infiltrate was examined for whole tumor sections in prior studies [23,31,34], we used tissue microarray sections that might not capture the CD8+ hotspots, and this might account for some of the discrepancy. Similar to our results, other studies have reported a significant association of the presence of MCPyV with tumoral PD-L1 expression and intratumoral CD8+ and FoxP3+ infiltrates [32,33,34]. In addition, Wardhani et al. [35] reported that higher tumoral IDO1 expression correlated with an MCPyV-positive status. Taken together, MCPyV infection appears to promote the expression of immune response-associated proteins, and correlates with better survival across various studies.

In our series, MCC-UP tumors (both virus-positive and -negative) were more immunogenic than their cutaneous counterparts. Of interest, the virus-positive UP group in our study appears to be the most immunogenic, with the highest frequency observed in the virus-positive UP group with respect to tumoral PD-L1 expression and in combination with high intratumoral CD8+ and FoxP3+ infiltrates. This immunoprofile observed in MCC-UPs is more in line with virus-positive MCC-KPs than virus-negative KPs. As noted in our and other studies, MCC-UP has a lower association with MCPyV than MCC-KP cases (25% versus 63%) [36]. Our findings support the role of immunotherapy in MCC-UP, which might include agents that block PD-1/PD-L1 axis and regulatory T-cell function or enhance T-cell activity. Several promising clinical trials of immune checkpoint inhibitors have demonstrated responses to anti-PD-L1 (avelumab, atezolizumab) and anti-PD1 (nivolumab, pembrolizumab) therapy [37]. In addition, a case of MCC-UP with a good response to avelumab has recently been reported [38]. 

Sequencing analyses by NGS and Sanger performed in our study showed that MCC-UP is comprised of two tumor groups with mutually exclusive segregation of UV or MCPyV pathways, as is described in recent studies of MCC-UP [15,16] and mirrors that in primary cutaneous MCCs [18,19,20,39]. In our series, a UV mutational signature with high TMB was noted in MCPyV-negative MCC-UPs and low TMB was noted in MCPyV-positive MCC-UPs, similar to findings reported by others [15,16]. Of the TMB-high cases or virus-negative MCCs, frequently mutated genes included *TP53*, *RB1*, and *PIK3CA* [18,19,20,21,39]. Similarly, molecular alterations of virus-negative MCC-UPs in our series involve TP53, RB1, the PI3K/AKT/mTOR pathway (*NF1*, *PIK3CA*, *PTEN*), *ARID1A*, and DNA repair genes (*ATM*, *BRCA2*). Our and prior studies suggested that it is likely that a combination of either high TMB or viral antigens and increased immunogenicity might account for the regression of primary tumors and an improved prognosis in MCC-UP cases [15,16].

Our study has several limitations, including its retrospective and multicenter nature, the small MCC-UP sample size, because of the rarity of MCC-UP, and only having 12 patients with stage III in the MCC-KP group. Due to the poor DNA quality of formalin-fixed paraffin-embedded tissues in older cases, we could perform NGS assays only on recent cases. Nevertheless, our study represents the first immunohistochemical comparison of the subgroups of MCC: virus-positive and virus-negative UP, and virus-positive and virus-negative KP groups.

## 4. Materials and Methods

The study was approved by Institutional Review Boards review of various institutions. A retrospective review of the pathology archives at six clinical institutions in Poland, Taiwan, and the United States from 1990 to 2020 yielded 134 primary cutaneous MCCs and 27 MCCs in lymph nodes of the parotid, axilla, and inguinal regions with no known primary. The diagnoses were histopathologically confirmed by the contributing pathologists and the corresponding author (MPH). All the tumors had diagnostic features of MCC, including characteristic histologic appearances and expression of CK20 and neuroendocrine markers (chromogranin, synaptophysin, neuron specific enolase, and/or CD56). Neurofilament, CK7, or other CKs (AE1/AE3, CAM5.2, pan or wide spectrum keratin), and EMA were assessed in a subset of cases. Thyroid transcription factor 1 (TTF1) was assessed in the majority of cases. Medical records, including clinical notes, pathology, and radiology reports, were reviewed to exclude the possibility of metastatic neuroendocrine carcinomas and to document clinical parameters, including age, lesion site, history of immunosuppression, stage, and disease status over time and at last follow-up (recurrence, metastasis) [40].

### 4.1. Immunohistochemistry

Tissue microarrays composed of two 2 mm tissue cores from each tumor were constructed. Immunohistochemical studies were performed on 5-micrometer-thick tissue sections using a Bond 3 automated immunostainer (Leica Microsystems, Bannockburn, IL, USA), with primary antibodies against MCPyV large T-antigen (CM2B4, sc-136172, 1:100, Santa Cruz Biotechnology, Dallas, TX, USA), TdT (SEN28, prediluted, Leica Microsystems), Pax5 (1EW, prediluted, Leica Microsystems), CK15 (EP14, prediluted, Bio SB, Goleta, CA, USA), CK19 (B170, prediluted, Leica Microsystems), p53 (D0-7, prediluted, Leica Microsystems), RB (1F8, prediluted, Bio SB), PD-L1 (E1L3N, 1:200, Cell Signaling Technology, Danvers, MA, USA), IDO1 (1F8.2, 1:400, Millipore, Burlington, MA, USA), CD8 (4B11, undiluted, Leica Microsystems), and FoxP3 (236A/E7, undiluted, BioCare Medical, Concord, CA, USA).

Scoring was done by two independent authors blinded to clinical outcomes. Tumor cells with positive CM2B4 nuclear staining of any intensity were considered positive. Scoring of CM2B4, TdT, Pax5, CK15, CK19, p63, p53, RB, and IDO1 immunostains was done using the *H*-score ((percentage at 1+) × 1 + (percentage at 2+) × 2 + (percentage at 3+) × 3), which combines the intensity and the percentage of positive cells into a combined score. The median *H*-scores of 20, 70, 190, 155, and 80 were used as cutoff values for TdT, Pax5, p53, Rb, and CK19, respectively. Greater than 1% of tumor cells with membranous PD-L1 staining was considered positive. Any staining with CM2B4 and CK15 was considered positive. The absolute cell counts of CD8+ and FoxP3+ lymphocytes within the tumor were assessed in three consecutive images captured at 40× magnification of the hotspot, the area with the densest labeling index. The scores were dichotomized into high and low using the median as the cutoff value.

### 4.2. Molecular Analyses

Molecular analyses for alterations involving tumor protein p53 (*TP53*), RB transcriptional corepressor 1 (*RB1*), and phosphatidylinositol-4,5-bisphosphate 3-kinase catalytic subunit alpha (*PIK3CA*) and the presence of MCPyV were performed on DNA extracted from 27 formalin-fixed paraffin-embedded MCC-UPs by polymerase chain reaction (PCR) and sequencing with specific primers using previously reported methods (Supplemental Table S5) [17,41].

NGS-based molecular analyses were successfully performed in 11 cases. The assay utilizes anchored multiplex polymerase chain reaction (PCR) to detect single nucleotide variants (SNV) and small insertion/deletions (indel) in genomic DNA using NGS (Supplemental Table S6). A sequencing library targeting hotspots and exons in 99 cancer genes was generated using two hemi-nested PCRs. Illumina MiSeq 2 × 151 base paired-end sequencing results were aligned to the hg19 human genome reference using BWA-MEM. MuTect and a laboratory-developed insertion/deletion analysis algorithm were used for SNV and indel variant detection, respectively.

### 4.3. Statistical Analyses

The statistical association between MCPyV T antigen expression (detected by the CM2B4 antibody) and other proteins and clinicopathologic features (age, gender, site, immunosuppression, stage, tumor size, tumor thickness, growth pattern, mitotic index, ulceration, necrosis, lymphovascular invasion, and perineural invasion) were evaluated by Fisher’s exact tests. American Joint Committee on Cancer stage versus MCPyV status was calculated using the Cochran–Armitage test. Overall survival (OS) was defined as the number of months from the initial diagnosis to the patient’s death by any cause. Kaplan–Meier plots and log-rank tests were created to visually assess the differences in the OS between subgroups. Box plots were used for the comparison of the expression of various immunostains. An assessment of the statistical significance of the differences between the groups was performed using Anova (for continuous expression) or the chi-squared test (for positive/negative markers). In a post-hoc analysis, Tukey’s test was applied to identify pairwise differences. Because of multiple testing, *p*-values were adjusted with the false discovery rate correction. All analyses and plots were performed using the R statistical package [42]. A two-tailed *p*-value of less than 0.05 was considered statistically significant.

## 5. Conclusions

In conclusion, we have comprehensively compared subtypes of MCC (UP and KP with respect to MCPyV status) and demonstrated significant immunophenotypic, immunologic, and molecular similarities between virus-negative MCC-UP and MCC-KP as well as virus-positive MCC-UP and MCC-KP. Our results showed distinct UV-signatures in MCPyV-negative tumors and high immunogenicity in MCPyV-positive tumors. Although additional studies are warranted for the MCPyV-positive cases, our findings are supportive of a cutaneous metastatic origin for MCPyV-negative MCC-UP tumors.

## Data Availability

The data presented in this study are available in this article and supplementary material.

## Supplementary Materials

The following are available online at https://www.mdpi.com/article/10.3390/cancers13071621/s1, Table S1: Summary of immunohistochemical results versus MCPyV status in 27 Merkel cell carcinomas of unknown primary; Table S2: Summary of clinicopathologic variables versus MCPyV status in primary Merkel cell carcinomas; Table S3: Summary of *p*-values of multiple comparisons in different tumor groups; Table S4: Results of Sanger and next generation sequencing (NGS); Table S5: Primers for polymerase chain reaction amplification and sequencing; Table S6: The gene targets covered by next generation sequencing analyses.
