# Supplementary material for: Merkel Cell Carcinoma of Unknown Primary: Immunohistochemical and Molecular Analyses Reveal Distinct UV-Signature/MCPyV-Negative and High Immunogenicity/MCPyV-Positive Profiles
Source: Cancers (Basel). 2021 Mar 31;13(7):1621. doi: 10.3390/cancers13071621 (PMC8037250; doi:10.3390/cancers13071621)
Supplement: Supplementary file 1 [file cancers-13-01621-s001.pdf]

cancers

MDPI

Article

Merkel Cell Carcinoma of Unknown Primary: Immunohistochemical and Molecular Analyses Reveal Distinct UV-Signature/MCPyV-Negative and High Immunogenicity/MCPyV-Positive Profiles.

Piotr Donizy, Joanna P. Wróblewska, Dora Dias-Santagata, Katarzyna Woznica, Przemyslaw Biecek, Mark C. Mochel, Cheng-Lin Wu, Janusz Kopczynski, Malgorzata Pieniazek, Janusz Ryś, Andrzej Marszalek and Mai P. Hoang

Supplementary Materials:

Table S1. Summary of immunohistochemical results versus MCPyV status in 27 Merkel cell carcinomas of unknown primary.

	MCPyV-Positive	MCPyV-Negative	*p*-Value
**Age**			
=> 70 years	7	8	**0.043 ***
< 70 years	1	11	
**Gender**			
Male	5	17	0.14
Female	3	2	
**Association with other cancer**			
Yes	2	5	1
No	6	14	
**Tumor size**			
> 2.5 cm	4	8	1
= < 2.5 cm	4	11	
**Tumoral PD-L1**			
> 1%	4	9	1
= < 1%	2	5	
**Tumoral IDO1**			
High	2	9	0.34
Low	4	5	
**Intratumoral CD8+ lymphocytes**			
High	5	10	1
Low	1	4	
**Intratumoral FoxP3+ lymphocytes**			
High	5	9	0.61
Low	1	5	
**TdT**			
High	2	1	0.2
Low	4	13	
**Pax5**			
High	2	3	0.61
Low	4	11	
**p53**			
High	2	12	**0.037 ***
low	4	2	
**Rb**			

High	2	0	0.078 .
Low	4	14	
	**CK15**		
High	0	9	**0.014** *
Low	6	5	
**CK19**			
High	3	12	0.13
Low	3	2	

* $p < 0.05$, statistically significant; . $p < 0.09$, approaching statistical significance.

Table S2. Summary of clinicopathologic variables versus MCPyV status in primary Merkel cell carcinomas.

	**MCPyV-Positive**	**MCPyV-Negative**	***p*-Value**
**Age**			
> 67 years	39	29	0.21
< = 67 years	45	21	
**Gender**			
Male	40	34	**0.031** *
Female	44	16	
**Immunosuppression**			
Present	7	6	0.55
Absent	77	44	
**AJCC stage at presentation**			
I	37	31	**0.021***
II	37	16	
III	10	2	
IV	0	0	
Not available	1	0	
**Site**			
Head and neck	33	32	**0.0073***
Other sites	51	18	
**Size**			
> 20mm	42	16	**0.048***
< = 20 mm	42	34	
**Thickness**			
> 10 mm	47	17	**0.012***
< = 10mm	35	33	
Not available	2	0	
**Ulceration**			
Present	23	21	0.09 .
Absent	61	29	
**Mitoses**			
> 40/ mm^2	38	29	0.21
<= 40/ mm^2	46	21	
**Growth pattern**			
Nodular	40	23	1
Nodular infiltrative, infiltrative	44	27	
**Necrosis**			
Present	31	11	0.088 .
Absent	53	39	
**Lymphovascular invasion**			
Present	38	26	0.48
Absent	46	24	
**Perineural invasion**			

Present	6	6	0.36
Absent	78	44	
**Epidermotropism**			
Present	1	10	**0.0002***
Absent	83	39	
**Associated keratinocytic neoplasms**			
Present	2	16	**0.0001***
Absent	82	34	
**Tumoral PD-L1 n=132**			
> 1%	48	16	**0.018***
= < 1%	37	31	
**Intratumoral CD8+ lymphocytes n=132**			
High	46	15	**0.018***
Low	39	32	
**Intratumoral FoxP3+ lymphocytes n=132**			
High	42	18	0.27
Low	43	29	
**Tumoral IDO1 n=130**			
High	45	18	0.11
Low	38	29	

* $p < 0.05$, statistically significant; $p < 0.09$, approaching statistical significance.

cancers

MDPI

Table S3. Summary of *p*-values of multiple comparisons in different tumor groups.

	TdT	Pax5	p53	Rb	CK15	CK19	IDO1	PD-L1	CD8	FoxP3	CD8-high FoxP3-high	PDL1-high FoxP3-high	PDL1-high CD8-high	PDL1-high IDO1-high
Virus+ UP vs virus- UP	0.99 (0.99)	0.71 (0.99)	**0.032** (0.097)	**0.0044 (0.0088)**	0.74 (0.96)	0.69 (0.99)	0.11 (0.22)	1 (1)	0.37 (0.37)	**0.0086 (0.017)**	0.84 (0.84)	1 (1)	0.84 (0.84)	1 (1)
Virus+ UP vs virus+ KP	0.23 (0.45)	0.83 (0.99)	0.92 (0.92)	0.36 (0.43)	0.92 (0.96)	0.99 (0.99)	0.17 (0.26)	0.95 (1)	0.13 (0.39)	**0.0015 (0.009)**	0.36 (0.54)	0.33 (0.40)	0.36 (0.54)	1 (1)
Virus+ UP vs virus- KP	0.98 (0.99)	0.99 (0.99)	0.41 (0.49)	**0.011 (0.016)**	0.44 (0.94)	0.92 (0.99)	0.86 (0.86)	0.24 (0.47)	0.066 (0.39)	**0.0061 (0.017)**	0.07 (0.19)	**0.034** (0.068)	**0.034** (0.068)	0.54 (0.88)
Virus- UP vs virus+ KP	**0.0085 (0.026)**	**0.014** (0.088)	**0.0016 (0.0096)**	**<0.0001 (<0.0001)**	0.86 (0.96)	0.14 (0.56)	0.85 (0.86)	0.79 (1)	0.97 (0.97)	1 (1)	0.62 (0.74)	0.28 (0.40)	0.62 (0.74)	0.59 (0.88)
Virus- UP vs virus- KP	0.8 (0.99)	0.41 (0.82)	0.12 (0.19)	0.74 (0.74)	0.96 (0.96)	0.82 (0.99)	0.062 (0.19)	0.068 (0.20)	0.78 (0.97)	0.97 (1)	0.094 (0.19)	**0.011** (0.066)	**0.04** (0.081)	0.0505 (0.15)
Virus+ KP vs virus- KP	**0.0022 (0.013)**	0.11 (0.33)	0.13 (0.19)	**<0.0001 (<0.0001)**	0.15 (0.91)	0.19 (0.56)	**0.021** (0.13)	**0.013** (0.079)	0.83 (0.97)	0.87 (1)	0.079 (0.19)	**0.033** (0.068)	**0.024** (0.081)	**0.048** (0.15)
Anova or Chi-square *p*-value	<0.001 ***	0.009 **	<0.001 ***	<0.001 ***	0.14	0.071	0.0042 **	0.028 *	0.094	0.0036 **	0.049 *	0.0075 **	0.014*	0.081

UP: unknown primary, KP: known primary; Asterisk indicates significant difference between group pairs: * $p < 0.05$; ** $p < 0.01$; *** $p < 0.001$. *p* values in parentheses: with false discovery rate correction.

Table S4. Results of Sanger and next generation sequencing (NGS).

Case	MCPyV	Sanger	Both Sanger and NGS	NGS

19 [1]	negative		PIK3CA p.Glu545Lys (c.1633G>A)	TP53 p.MetGly243IleSer (c.729_730delGGinsAA)<br>RB1 splice acceptor variant (c.1390-1G>A)<br>RB1 splice acceptor(c.1696-1_1696delGGinsAA)<br>APC p.Asn1815Asp (c.5443A>G)<br>ARID1A p.Pro699Ser (c.2095C>T)<br>ATM p.Arg248Ter (c.742C>T)<br>CIC splice region variant (c.4035-7C>T)<br>GNAQ splice acceptor variant (c.477-1G>A)<br>STK11 p.Phe354Leu (c.1062C>G)<br>TSC1 splice region variant (c.1030-6T>G)
18	negative	TP53 p.Pro82Ser, c.244C>T		TP53 p.Arg342Ter (c.1024C>T)<br>TP53 p.Leu330Pro (c.989T>C)<br>RB1 p.Gln504Ter (c.1510C>T)<br>RB1 loss<br>BRCA2 p.Pro2036Leu (c.6106_6107delCCinsTT)<br>EGFR p.Glu114Lys (c.340G>A)<br>MYC p.Glu8Lys (c.22G>A)<br>NF1 p.LeuLeu1892PheIle (c.5676_5677delACinsTA)
17	negative	TP53 p.Pro87Leu, c.260C>T	TP53 p.Glu286Lys (c.856G>A)	TP53 p.Pro250Phe (c.748_749delCCinsTT)<br>RB1 p.Arg251Ter (c.751C>T)<br>ARID1A p.Pro2095Ser (c.6283C>T)<br>BRCA2 splice region variant (c.476-7_476-6delCCinsTT)<br>FGFR2 p.Ser799Thr (c.2395T>A)<br>KDR p.Glu1203Val (c.3608A>T)
16	negative			TP53 p.Arg342Ter (c.1024C>T)<br>TP53 p.Gly245Asp (c.734G>A)<br>RB1 splice acceptor variant (c.1128-1_1128delGGinsAA)<br>APC p.Pro1268Ser (c.3802C>T)<br>ARID1A p.Pro580Ser (c.1738C>T)<br>HRAS p.Gly12Val (c.35G>T)<br>MAP3K1 p.Pro373Ser (c.1117C>T)<br>NF1 p.Asn1984Ser (c.5951A>G)<br>PTCH1 p.Gly1296Arg (c.3886G>A)<br>STAG2 splice region variant (c.1117-5C>T)<br>TSC1 p.Lys918Ter (c.2752A>T)

8 [1,2]	negative	TP53 p.Pro98Leu, c.203C>T<br>PIK3CA p.Thr208Ile, c.623C>T	PIK3CA p.Glu545Lys (c.1633G>A)	TP53 p.Glu286Lys (c.856G>A)<br>RB1 splice donor variant (c.2489+1G>A)<br>GNAS c.756G>A(p.=) (c.756G>A)<br>NOTCH1 p.Ser2198Leu (c.6593C>T)
108 [2]	negative	PIK3CA p.Pro200Ser, c.598C>T	RB1 p.Trp681Ter (c.2043G>A)	TP53 splice donor variant (c.375+1G>A)<br>RB1 splice acceptor variant (c.1499-1_1499delGGinsAA)<br>ARID1A p.His1960Tyr (c.5878C>T)<br>BRAF p.Arg462Lys (c.1385G>A)<br>CIC splice region variant (c.218-6C>T)<br>CIC p.Asp449Asn (c.1345G>A)<br>RET c.1761G>A(p.=) (c.1761G>A)<br>PTEN splice acceptor variant (c.493-1G>A)<br>TERT promoter variant (hg19 chr5:g.1295250C>T; c.-146C>T; C250T)<br>TSHR p.His248Asn (c.742C>A)
24 [1]	positive	TP53 p.Trp53*, c.159G>A		TSC1 splice region variant (c.364-7C>T)
9	positive	TP53 p.Gln100*, c.298C>T<br>PIK3CA p.Glu85Lys, c.253G>A		TSC2 p.Trp167Arg (c.499T>C)
23 [1]	positive			
174	positive			
7 [1]	positive		PIK3CA p.His1047Leu (c.3140A>T)	
15	negative	TP53 p.Ser260Pro, c.778T>C<br>TP53 p.Gln317Serfs*28, c.949del<br>PIK3CA p.Glu542Lys, c.1624G>A		
14	negative	TP53 p.Arg248Trp, c.742C>T<br>PIK3CA p.Arg115Gln, c.344G>A		
13	negative			
11	negative	TP53 p.Pro77Leu, c.230C>T<br>RB1 p.Arg876His,c.2627G>A		

		PIK3CA p.Gln75*, c.223C>T
26	negative	TP53 p.Leu252Pro, c.755T>C<br>TP53 p.Glu286Lys, c.856G>A<br>PIK3CA p.Lys227Glu, c.679A>G
21	negative	
20	negative	TP53 p.Glu294*, c.880G>T<br>RB1 p.Arg376_Val378delinsSer, c.1128_1133del
106	negative	TP53 p.Arg213*, c.637C>T
167	negative	PIK3CA p.Glu545Lys, c.1633G>A
168	negative	TP53 p.Val147Ala, c.440T>C<br>TP53 p.Pro152Ser, c.454C>T
170	negative	TP53 p.Thr102Ile, c.305C>T<br>TP53 p.Pro151Leu, c.452C>T<br>TP53 p.Ser241Thr, c.721T>A<br>RB1 p.Trp195*, c.585G>A
171	negative	TP53 p.Gly245Ser, c.733G>A<br>PIK3CA p.Glu545Lys, c.1633G>A
172	negative	TP53 p.Pro151Phe, c.451_452delinsTT
25	positive	
22	positive	RB1 p.Arg798Trp, c.2392C>T<br>RB1 p.Ser882Leu, c.2645C>T
173	positive	

[1] Older archival formalin-fixed paraffin-embedded samples yielded lower quality nucleic acid and poor sequencing diversity on the NGS platform, which may affect detection of some variants. [2] *PIK3CA* exon 4 (including *PIK3CA* codons Pro200 and Thr208) was probed by Sanger sequencing but is not targeted by the NGS sequencing panel.

Table S5. Primers for polymerase chain reaction amplification and sequencing.

Gene	Forward	Reverse	Amplicon
TP53 exon 9	GGAGACCAAGGGTGCAGTTA	CCCCAATTGCAGGTAAAACA	232
TP53 exon 8	GGGACAGGTAGGACCTGATTT	GCATAACTGCACCCTTGGTC	248
TP53 exon 7	TGCTTGCCACAGGTCTCC	GGTCAGAGGCAAGCAGAGG	236
TP53 exon 6	GAGAGACGACAGGGCTGGT	CACTGACAACCACCCTTAACC	231
TP53 exon 5	GTTTCTTTGCTGCCGTCTTC	GGGCCAGACCTAAGAGCAAT	357
TP53 exon 4	CCTGGTCCTCTGACTGCTCT	GCCAGGCATTGAAGTCTCAT	362
PIK3CA exon2	ATGCCCCCAAGAATCCTAGT	CGAAGGTATTGGTTTAGA-CAGAAA	400
PIK3CA exon10	CTGTGAATCCAGAGGGGAAA	AACAGA-GAATCTCCATTTTAGCA	217
PIK3CA exon4	GGTGATTGCATCTAATGTTTTCC	CCTGGGCGAGAGTGAGATT	393
PIK3CA exon6	CGAGTGTGTGCATATGTG-TATGTT	GCCCAGGCTGGTCTAAAAA	244
PIK3CA exon21	CTCAATGATGCTTGGCTCTG	TGGAATCCAGAGTGAGCTTTC	241
RB1 exon 3	TGTTATGTGCAAAC-TATTGAAACAAG	TTTGAAGTTGTTTTTAAAATGA-GAA	276
RB1 exon 6	GCACAAAAAGAAACACCCAAA	GTCCAAAGGAATGCCAATTT	230
RB1 exon 8	GAATGTTACCAAGATTATTTTT-GACC	TTTAACTGTTTTAAA-GAAATCATGAAG	282
RB1 exon 12	AGACAAGTGGGAGGCAGTGT	CAAGTTTCTTTGCCAAGA-TATTACA	260
RB1 exon 20	GCGATTTCATGATTTGAAAAA	GGGAGGAGAGAAGGTGAAGTG	260
RB1 exon 23	AATGGGTCCACCAAAACATT	GGATCAAAATAATCCCCCTCTC	275
RB1 exon 25	TGCCTGATTTTTGACACACC	ATGACCATCTCGGCTACTGG	285
MCVPS1	TCAGCGTCCCAGGCTTCAGA	TGGTGGTCTCCTCTCTTGC-TACTG	109
MCPyV	ACTTGGGAAAGTTTT-GACTGGTGGCAA	GGGCCTCGTCAACCTAGATGG-GAAAG	195
LT4	TTCCTCTGGGTATGGGTCCTT	GGTCCTCTGGACTGGGAGTCT	

Table 6. The gene targets covered by next generation sequencing analyses are as follows:.

The single nucleotide variant and indel gene targets covered by this test are as follows (exons): ABL1 (4-7), AKT1 (3,6), ALK (21-23,25), APC (16), ARID1A (1-20), ATM (1-63), ATRX (1-35), AURKA (2,5-8), BRAF (11,15), BRCA1 (2-23), BRCA2 (2-27), CCNB1 (2,[3-partial],5,[6-partial],7), CCND2 ([2-partial],3-4,[5-partial]), CCND3 (2-5-partial), CCNE1 (3-8,10,12), CDH1 (1-16), CDK4 (2-7), CDK6 (6), CDKN2A (1-3), CIC (1-20), CSF1R (7,22), CTNNB1 (3), DAXX (1-8), DDR2 (12-18), DDX3X (1-17), EGFR (3,7,15,18-21), ERBB2 (8,10,19-21,24), ERBB3 (2-3,7-8), ERBB4 (3-4,6-9,15,23), ESR1 (8), EZH2 (16), FBXW7 (1-11), FGFR1 (4,7-8,13,15,17), FGFR2 (7,9,12,14), FGFR3 (7-9,14-16,18), FLT3 (11,14,16,20), FOXL2 (1), GNA11 (5), GNAQ (4-5), GNAS (6-9), H3F3A (2), HNF1A (3-4), HRAS (2-3), IDH1 (3-4), IDH2 (4), JAK2 (11,13-14,16,19), JAK3 (4,13,16), KDR (6-7,11,19,21,26-27,30), KEAP1 (2-6), KIT (2,8-11,13-15,17-18), KRAS (2-5), MAP2K1 (2,3,6-7), MAP3K1 (1-20), MDM2 (2-4,6,8,10), MDM4 ([4-partial],5-6,[7,9-11-partial]), MEN1 (2-10), MET (2,11,14,16,19,21), MITF (1-partial), MLH1 (12), MPL (10), MSH6 (1-10), MSI, MYC (1-3), MYCN (3), NF1 (1-58), NF2 (1-15), NKX2-1 (1-partial), NOTCH1 (25-27,34), NPM1 (11), NRAS (2-5), PDGFRA (12,14-15,18,23), PIK3CA (2,5,7-8,10,14,19,21), PIK3R1 (1-10), POLE (9-14), PTCH1 (1-23), PTEN (1-9), PTPN11 (3,13), RB1 (1-27), RET (10-11,13-16), RHOA (2-3), RNF43 (2-10), ROS1 (36-38), SDHB (1-8), SMAD2 (7), SMAD4 (2-12), SMARCA4 (3-36), SMARCB1 (2,4,5,9), SMO (3,5-6,9,11), SRC (14), STAG2 (3-34), STK11 (1-9), SUFU (1-12), TERT (1), TP53 (1-11), TP63 (1-14), TSC1 (3-23), TSC2 (2-42), TSHR (10), VHL (1-3).

The copy number variant gene targets covered by this test are as follows: ABL1, AKT1, ALK, APC, ARID1A, ATM, ATRX, AURKA, BRAF, BRCA1, BRCA2, CAMTA1, CCNB1, CCND1, CCND2, CCND3, CCNE1, CDK4, CDKN2A, CDK6, CIC, CDH1, CSF1R, DAXX, DDR2, DDX3X, EGFR, ERBB2 (HER-2), ERBB3, ERBB4, FBXW7, FGF19, FGFR1, FGFR2, FGFR3, FLT3, FOXL2, GLI2, GNA11, GNAQ, GNAS, HNF1A, HRAS, IDH1, JAK2, JAK3, KDR, KEAP1, KIT, KRAS, MAP2K1, MAP3K1, MDM2, MDM4, MEN1, MET, MITF, MLH1, MSH6, MYC, MYCN, NF1, NF2, NKX2-1, NOTCH1, NRAS, PDGFRA, PIK3CA, PIK3R1, PLAUR, POLE, PTCH1, PTEN, PTPN11, RB1, RET, RHOA, RNF43, SDHB, SMAD2, SMAD4, SMARCA4, SMARCB1, SMO, SRC, STAG2, STK11, SUFU, TERT, TP53, TP63, TSC1, TSC2, and VHL.
